# Relationship Between Intragastric Meal Distribution, Gastric Emptying, and Gastric Neuromuscular Dysfunction in Chronic Gastroduodenal Disorders

**DOI:** 10.1111/nmo.70170

**Published:** 2025-09-22

**Authors:** Chris Varghese, Armen A. Gharibans, Daphne Foong, Gabriel Schamberg, Stefan Calder, Vincent Ho, Reena Anand, Christopher N. Andrews, Alan H. Maurer, Thomas Abell, Henry P. Parkman, Greg O'Grady

**Affiliations:** ^1^ Department of Surgery University of Auckland Auckland New Zealand; ^2^ Department of Surgery Mayo Clinic Rochester Minnesota USA; ^3^ Perelman School of Medicine University of Pennsylvania Philadelphia Pennsylvania USA; ^4^ Western Sydney University Sydney New South Wales Australia; ^5^ Alimetry Ltd Auckland New Zealand; ^6^ Department of Medicine Temple University Hospital Philadelphia Pennsylvania USA; ^7^ Department of Gastroenterology University of Calgary Calgary Alberta Canada; ^8^ Division of Gastroenterology, Hepatology and Nutrition University of Louisville Louisville Kentucky USA

**Keywords:** body surface gastric mapping, dyspepsia, gastroparesis, intragastric meal distribution, scintigraphy

## Abstract

**Background:**

Chronic gastroduodenal symptoms arise from heterogeneous gastric motor dysfunctions. This study applied multimodal physiological testing using gastric emptying scintigraphy (GES) with intragastric meal distribution (IMD) and Gastric Alimetry body surface gastric mapping (BSGM) to define motility and symptom associations.

**Methods:**

Patients with chronic gastroduodenal symptoms underwent simultaneous supine GES and BSGM with a 30 m baseline, 99mTC‐labeled egg meal, and 4 h postprandial recording. IMD (ratio of counts in the proximal half of the stomach to the total gastric counts) was calculated immediately after the meal (IMD0), with < 0.568 defining abnormal IMD. BSGM phenotyping followed a consensus approach, based on normative spectral reference intervals.

**Results:**

Among 67 patients (84% female, median age 40 years, median BMI 24 kg/m^2^), median IMD0 was 0.76 (IQR: 0.69–0.86) with 5 (7.5%) meeting abnormal IMD criteria. Delayed gastric emptying (*n* = 18) was associated with higher IMD0 (median 0.9 vs. 0.7, *p* = 0.004). On BSGM, 15 patients had abnormal spectrograms (5 [7.5%] high frequency and 10 [14.9%] low rhythm stability and/or amplitude); and in these patients, higher IMD0 (proximal retention) strongly correlated to delayed BSGM meal responses (*R* = −0.71, *p* = 0.003). Lower IMD, indicating antral distribution, correlated with higher gastric frequencies (*R* = −0.27, *p* = 0.03). BSGM abnormalities paired with abnormal IMD were associated with worse dyspeptic symptoms.

**Conclusion:**

Proximal retention of food as assessed by IMD correlated with delayed emptying, and in the presence of neuromuscular spectral abnormalities (abnormal frequencies or rhythms), delayed motility responses on BSGM. Patients with multiple motor abnormalities experience worse dyspeptic symptoms.

## Graphical abstract

Multimodal physiological testing using gastric emptying scintigraphy with intragastric meal distribution and body surface gastric mapping showed proximal retention of food correlated with delayed emptying and neuromuscular spectral abnormalities (abnormal slow wave frequencies or rhythms) and delayed motility responses on BSGM. Patients with multiple motor abnormalities experience worse dyspeptic symptoms.

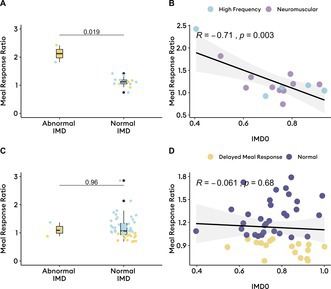

**Summary**

- Combined physiological testing using gastric emptying scintigraphy with intragastric meal distribution analysis, and body surface gastric mapping provides complementary insights into gastric motor function.
- Proximal retention observed on intragastric meal distribution analysis is associated with delayed gastric emptying.
- Meal responses are most sensitive to alterations in distribution among patients with abnormal myoelectrical activity.

## 1 Introduction

Chronic neurogastroduodenal disorders arise from heterogeneous mechanisms, with some patients demonstrating motor dysfunction and others seemingly lacking motility abnormalities. While a range of motility abnormalities, such as impaired accommodation, antral hypomotility, and pyloric dysfunction, are implicated in chronic gastroduodenal disorders [1, 2], non‐motility‐related factors such as inflammation, visceral hypersensitivity, hormonal feedback loops, and centrally mediated mechanisms also contribute [3]. Robustly defining these features may help clarify diagnostic classifications and guide personalized therapies.

Gastric emptying testing has been the most widely used test of gastric function, with scintigraphy being the gold standard for evaluating gastric transit. However, gastric emptying status correlates poorly with symptoms and can be labile over time [4, 5]. In addition to summative measures of transit, scintigraphic assessment of gastric emptying can allow evaluation of meal distributions when advanced analysis methods are applied [6]. Impairments of intragastric meal distributions (IMD) measured in this way have previously been implicated in functional dyspepsia [7, 8].

Recently, Gastric Alimetry has emerged as a new noninvasive test of gastric myoelectrical activity, enabling robust evaluation of the myoelectrical coordination of gastric motility [9, 10], and the dynamic postprandial meal response [11]. In addition, validated time‐of‐test symptom profiling aids comparison of physiological abnormalities as they pertain to symptom genesis [10, 12].

The aim of this study was therefore to apply multimodal physiological profiling using gastric emptying scintigraphy (GES) with IMD together with Gastric Alimetry body surface gastric mapping (BSGM) to comprehensively define motility and symptom associations in patients with chronic gastroduodenal disorders.

## 2 Methods

This was a prospective observational cohort study conducted in Auckland, New Zealand, and Western Sydney, Australia. Ethical approvals were granted at each institution (AHREC123; H13541), and all patients provided informed consent. The study is reported in accordance with the STROBE statement [13].

### 2.1 Inclusion Criteria

Consecutive patients aged ≥ 18 years with chronic gastroduodenal symptoms consistent with chronic nausea and vomiting syndromes, functional dyspepsia, and/or gastroparesis, with a negative upper gastrointestinal endoscopy undergoing GES were invited to participate. Functional dyspepsia and chronic nausea and vomiting syndromes were diagnosed using Rome IV criteria in the absence of a delayed gastric emptying [14]. Exclusion criteria included those with known structural gastrointestinal diseases and previous abdominal surgery. Patients with cannabinoid hyperemesis were also excluded. Specific exclusion criteria for Gastric Alimetry including a BMI of > 35, active abdominal wounds or abrasions, fragile skin, and allergies to adhesives were also applied.

### 2.2 Physiological Testing

Patients underwent simultaneous Gastric Alimetry (Alimetry, New Zealand) and GES testing using standardized test protocols [10, 15]. Tests were conducted after an overnight fast, and medications affecting GI motility (e.g., opiates) were withheld for 48 h prior to testing. Patients were asked to avoid caffeine, nicotine, and cannabis 48 h before testing. Selective serotonin reuptake inhibitors were withheld on the morning of testing. Glucose levels were controlled in diabetic subjects. The standardized ~255 kcal low‐fat egg meal radiolabeled with ^99m^Tc was used, as previously reported [6, 15, 16]. The egg meal is known to elicit the same meal response as the standard Gastric Alimetry meal; however, it is noted that amplitude and the Gastric Alimetry Rhythm Index (GA‐RI) can be lower than seen with the larger standard Gastric Alimetry oatmeal and nutrient drink (482 kcal) meal [17].

#### 2.2.1 Gastric Emptying Scintigraphy

GES images were acquired at 0, 1, 2, and 4 h after meal ingestion while patients were in a supine position. Delayed gastric emptying was present if gastric retention was > 10% at 4 h [15]. IMD is the ratio of counts in the proximal half of the stomach to the total gastric counts, which was calculated immediately after the meal (time, 0 min; IMD0). The regional IMD was assessed using the methods of Parkman et al. [18], with the proximal and distal halves of the stomach equally divided along the midpoint of the longitudinal axis of the stomach as previously described by Orthey et al. and Piessevaux et al. [6, 19] For discrimination of abnormal IMD, < 0.568 was used as the cut‐off, as previously determined on receiver‐operating‐characteristic analysis [6].

#### 2.2.2 Gastric Alimetry

BSGM was performed using the Gastric Alimetry system, which includes a high‐resolution stretchable electrode array (8 × 8 electrodes; 20 mm inter‐electrode spacing; 196 cm^2^), a wearable Reader, and an iPadOS App for concurrent validated symptom logging during the test [9, 12, 20]. Array placement was preceded by shaving if necessary, and skin preparation (NuPrep; Weaver & Co, CO, USA). Recordings were performed simultaneously with GES encompassing a 30 min fasting baseline, a 10 min meal, and a 4 h postprandial recording. Patients sat in a reclined relaxed position with limited movement, then transferred to the nuclear medicine table for imaging, with motion artifacts automatically corrected or rejected using validated algorithms [21]. Symptoms including early satiation, nausea, bloating, upper gut pain, heartburn, stomach burn, and excessive fullness were measured during continuous testing at 15‐min intervals using 0–10 visual analogue scales (0 indicating no symptoms; 10 indicating the worst imaginable extent of symptoms) and combined to form a “Total Symptom Burden Score.” [12] The Total Symptom Burden Score is a validated score employed with time‐of‐test gastroduodenal diagnostic tests that shows convergent and concurrent validity against PAGI‐SYM and PAGI‐QOL metrics [12].

BSGM spectral analysis included Principal Gastric Frequency (PGF; reference intervals: 2.65–3.35 cycles per minute), BMI‐adjusted amplitude (reference intervals: 22–70 μV), and Gastric Alimetry Rhythm Index (GA‐RI; reference intervals: ≥ 0.25) as previously described [22]. In addition, the ‘meal response ratio’ (MRR) was used to characterize meal response timing; calculated as the ratio of the average amplitude in the first 2 h postprandially to that of the last 2 h [11]. MRR was not calculated if postprandial recording duration was < 4 h. A normal MRR was empirically defined as > 1 based on previous studies [9, 22, 23], meaning that the dominant gastric motor response occurred within the first 2 h after a meal. Phenotyping was based on an established consensus hierarchical approach: [24]

- Neuromuscular phenotype: GA‐RI < 0.25 and/or BMI‐adjusted amplitude < 22.
- High frequency phenotype: PGF > 3.35 cycles per minute.
- Delayed meal response phenotype: MRR < 1 (considered normal spectrogram per current normative reference interval criteria) [22].
- Normal phenotype: GA‐RI, BMI‐adjusted amplitude, and PGF within normative reference intervals.

In this analysis, spectral data were considered “abnormal” if the subject had a high frequency or neuromuscular phenotype. Neuromuscular dysfunction, as used here, refers to abnormal rhythm or amplitude of the BSGM signal, which reflects a combination of gastric slow waves and gastric contractility [9]. Neuromuscular function, and particularly coordination of gastric slow waves, is expected to be impaired in the setting of interstitial cell of Cajal depletion [25, 26]. The term gastric motor dysfunction refers to any abnormality of gastric slow waves and resultant gastric motility, including rhythm stability, amplitude, frequency, or timing (as measured by the spectral metrics of GA‐RI, BMI‐adjusted amplitude, PGF, and MRR).

### 2.3 Data Analysis

All analyses were performed in Python v3.9.7 and R v.4.4.2 (R Foundation for Statistical Computing, Vienna, Austria). Continuous data were summarized as mean (standard deviation) or median (interquartile range) based on visual and statistical evaluation for normality, with appropriate tests for parametric or nonparametric data performed. Assumptions for correlation tests were assessed using the *performance* package, with Spearman or Pearson coefficients reported accordingly [27]. Categorical data were cross‐tabulated, and differences tested using *χ* ^2^ tests. To assess differential symptom impacts by results of multimodal physiological testing, a linear regression analysis was performed with interaction terms between Gastric Alimetry phenotypes and accommodation status based on IMD0. Results of linear regression are reported as *β* coefficient and 95% confidence intervals (CI).

## 3 Results

Sixty‐seven patients underwent simultaneous BSGM and GES with sufficient data for meal distribution analysis (19 [28.4%] in Auckland and 48 [71.6%] in Western Sydney). Median age was 40 (IQR: 28–56), 56 (84%) were female, and median BMI was 24 [9, 23, 24, 25, 26, 27, 28, 29]. The cohort is summarized in Table 1. On gastric emptying testing, median T1/2 was 54.5 (IQR 37.6–89.4), median percentage retained at 4 h was 3% (IQR 0.7–11) with 18 (26.9%) meeting criteria for delayed emptying. The median IMD0 was 0.76 (IQR 0.69–0.86) with 5 (7.5%) classified as having abnormal IMD. Gastric Alimetry phenotyping showed 10 (14.9%) had a neuromuscular phenotype (low rhythm stability and/or amplitude), 5 (7.5%) had a high frequency, 18 (26.9%) had a delayed MRR, and 34 (50.7%) had a normal phenotype.

**TABLE 1:** Demographic and physiological parameters stratified by accommodation status.

		Abnormal IMD	Normal IMD	Total	*p*
Age	Median (IQR)	45.0 (32.0 to 54.0)	39.0 (27.2 to 55.8)	40.0 (27.5 to 55.5)	0.65
Sex	Female	2 (40.0)	54 (87.1)	56 (83.6)	0.035
	Male	3 (60.0)	8 (12.9)	11 (16.4)	
BMI	Median (IQR)	22.9 (22.5 to 27.1)	24.5 (22.6 to 29.8)	24.4 (22.6 to 29.8)	0.685
Practice	Auckland	2 (40.0)	17 (27.4)	19 (28.4)	0.933
	Western Sydney	3 (60.0)	45 (72.6)	48 (71.6)	
IMD0 (%)	Median (IQR)	0.5 (0.4 to 0.5)	0.8 (0.7 to 0.9)	0.8 (0.7 to 0.9)	< 0.001
T1/2	Median (IQR)	50.5 (28.8 to 61.7)	55.2 (38.1 to 94.4)	54.5 (37.6 to 89.4)	0.328
Percentage retained at 4 h	Median (IQR)	0.5 (0.3 to 0.7)	3.1 (0.8 to 11.3)	3.0 (0.7 to 10.9)	0.097
GES status	Delayed	1 (20.0)	17 (27.4)	18 (26.9)	1
	Normal	4 (80.0)	45 (72.6)	49 (73.1)	
GA‐RI	Median (IQR)	0.5 (0.5 to 0.6)	0.5 (0.3 to 0.6)	0.5 (0.3 to 0.6)	0.489
PGF	Median (IQR)	3.2 (3.1 to 3.3)	3.0 (2.9 to 3.2)	3.0 (2.9 to 3.2)	0.202
BMI‐Adjusted Amplitude	Median (IQR)	32.1 (30.8 to 44.7)	34.3 (27.7 to 41.1)	34.3 (27.7 to 41.4)	0.867
Meal response ratio	Median (IQR)	1.4 (1.1 to 1.8)	1.1 (0.9 to 1.3)	1.1 (1.0 to 1.4)	0.215
Gastric Alimetry phenotype	Delayed meal response	1 (20.0)	17 (27.4)	18 (26.9)	0.694
	High frequency	1 (20.0)	4 (6.5)	5 (7.5)	
	Neuromuscular	1 (20.0)	9 (14.5)	10 (14.9)	
	Normal	2 (40.0)	32 (51.6)	34 (50.7)	

Abbreviations: BMI, body mass index; GA‐RI, Gastric Alimetry Rhythm Index; GES, gastric emptying scintigraphy; IMD0, intragastric meal distribution at time = 0; IQR, interquartile range; PGF, principal gastric frequency.

### 3.1 Multimodal Physiological Assessment

**FIGURE 1:**
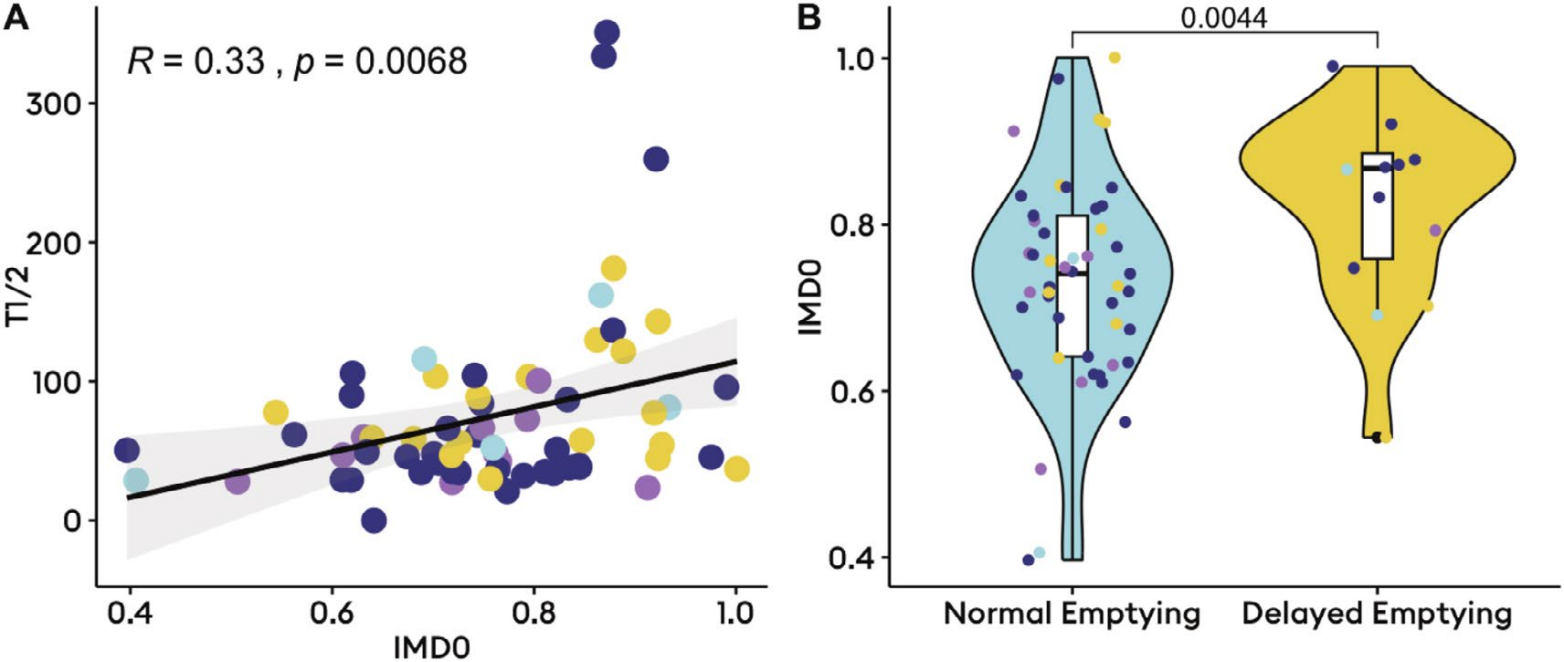
Relationship between gastric emptying scintigraphy transit delays and intragastric meal distribution. (A) Correlation between T1/2 and intragastric meal distribution at time 0 (IMD0); (B) Violin and box plots depicting the median, interquartile range, range and distribution of IMD0 by emptying status. Colored dots represent phenotype (yellow, delayed meal response; light blue, high frequency; purple, neuromuscular; purple, normal). IMD0, intragastric meal distribution at time 0; T1/2, half emptying time.

**FIGURE 2:**
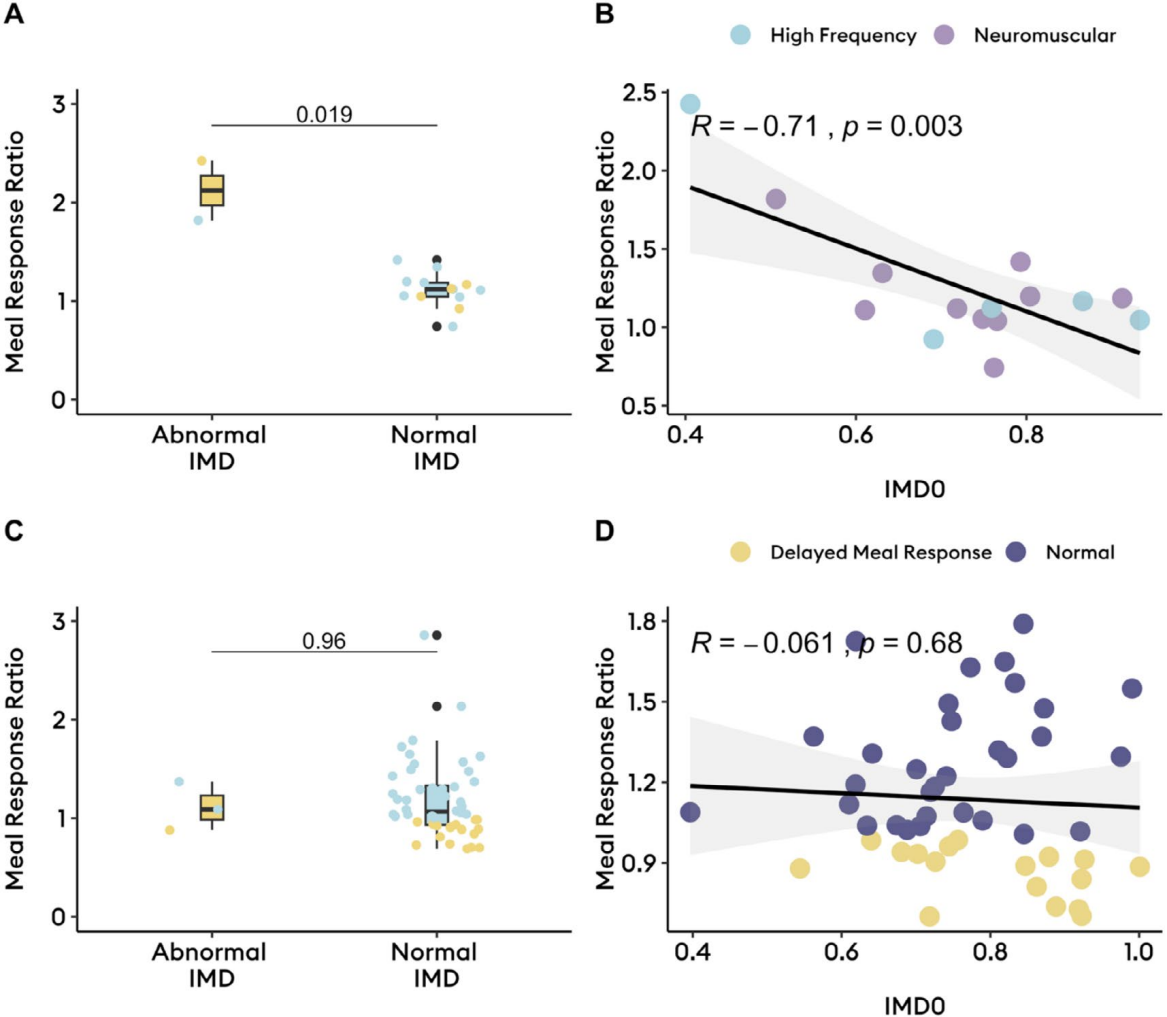
Impact of meal distribution on meal response ratios and differential impacts by neuromuscular function. (A, B) In those with abnormal spectrograms (*n* = 18), Early antral redistribution was associated with earlier meal responses (*p* < 0.02); (C, D) In those with normal spectrograms (*n* = 52), meal distribution was not associated with meal response timing (*p* > 0.65). IMD0, intragastric meal distribution at time 0.

**FIGURE 3:**
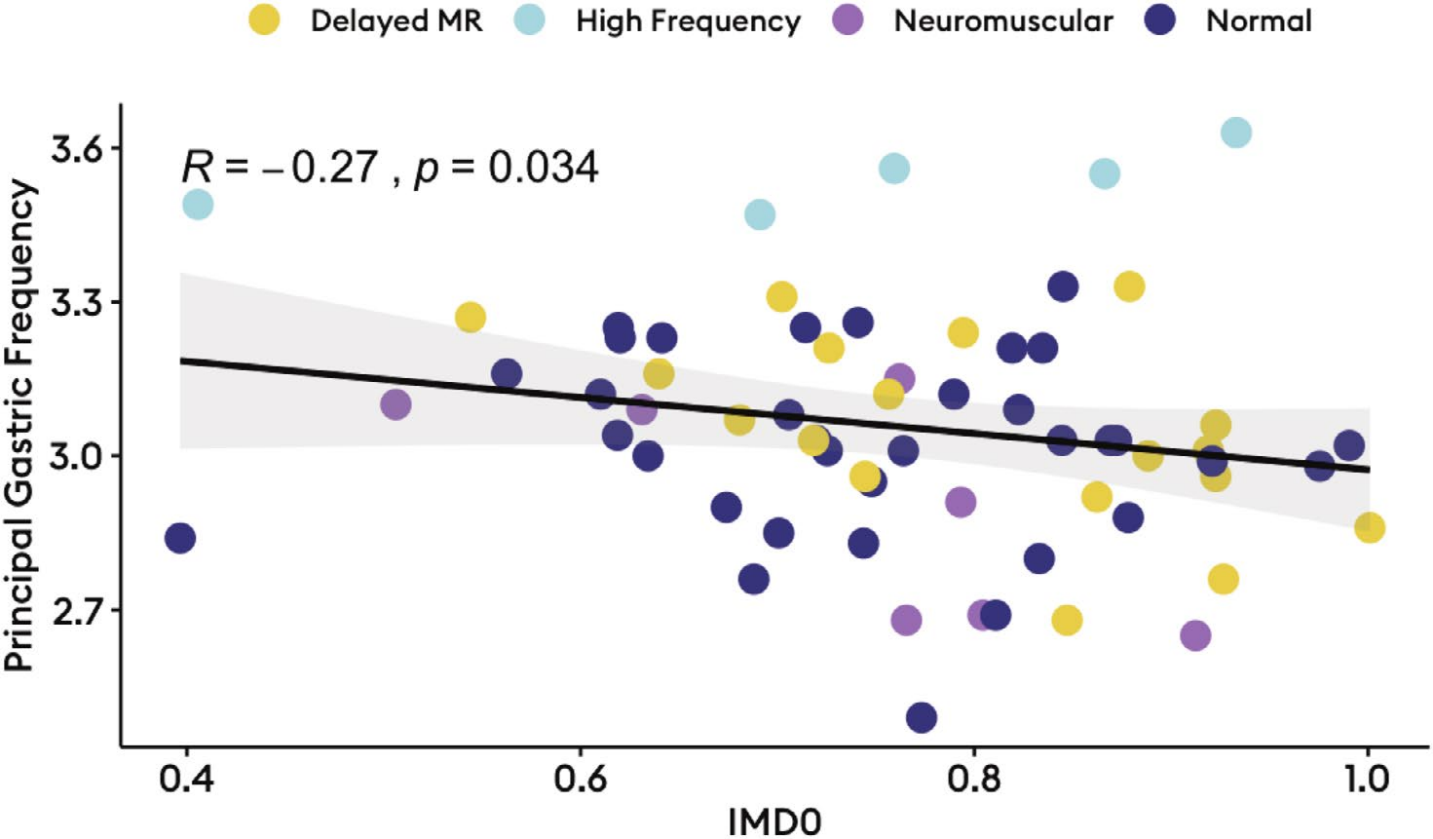
Correlation between frequency and intragastric meal distribution, with dots colored by Gastric Alimetry phenotype.

Delayed gastric emptying (*n* = 18) was associated with higher IMD0 (median 0.9 [IQR: 0.8–0.9] vs. 0.7 [IQR: 0.6–0.8], *p* = 0.004), indicating greater proximal retention after meal ingestion. Higher IMD0 also correlated with slower gastric emptying T1/2 (Pearson *R* = 0.33, *p* = 0.007; Figure 1). Delayed gastric emptying was also associated with higher rhythm stability (median 0.6 [IQR: 0.5–0.6] vs. 0.5 [0.3–0.6], *p* = 0.04), but otherwise comparable spectral metrics on BSGM (*p* > 0.05; Table S1). There was no significant difference in IMD0 between the different Gastric Alimetry phenotypes (*p* = 0.4; Figure S1). Among the 15 patients that had abnormal spectrograms (5 [7.5%] with high frequencies; 10 [14.9%] with low rhythm stability and/or amplitude), high IMD0 (proximal retention) was strongly correlated to delayed BSGM meal‐response timing (Pearson *R* = −0.71, *p* = 0.003; Figure 2), meaning proximal retention was associated with a delayed onset of gastric motor activity. This relationship was not seen among those with normal spectrograms (Figure 2C,D). Additionally, lower IMD, indicating antral distribution, was correlated with higher gastric frequencies (Spearman *R* = −0.27, *p* = 0.03; Figure 3).

### 3.2 Symptom Associations

Patients with a delayed meal response on BSGM had higher excessive fullness than those with a normal phenotype (mean difference 2.0, *p* = 0.05). In addition, patients with a neuromuscular phenotype had lower heartburn symptoms than those with a normal phenotype (mean difference: −2.1, *p* = 0.04). All univariable symptom comparisons are reported in Table S2 and Figure S2. Using a linear regression model, evaluating IMD status by BSGM phenotype showed that the group with abnormal IMD on average had lower total symptom burden (*β* = −17, 95% CI: −12 to −10, *p* < 0.001); however, when abnormal IMD was paired with a physiological abnormality on BSGM, total symptom burden was higher relative to those with normal IMD and BSGM (*β* > 10, *p* < 0.03; Table 2). Specifically, the neuromuscular phenotype paired with abnormal IMD was associated with worse stomach burn (*β* = 1.6, 95% CI: 0.3–3.0, *p* = 0.02) and excessive fullness (*β* = 3.1, 95% CI: 0.5–5.6, *p* = 0.02); and the high frequency phenotype paired with abnormal IMD was associated with worse bloating (*β* = 4.5, 95% CI: 2.3–6.8, *p* < 0.001), excessive fullness (*β* = 3.8, 95% CI: 0.5–7.1, *p* = 0.03), and stomach burn (*β* = 4.4, 95% CI: 2.6–6.1, *p* < 0.001). Delayed meal responses paired with abnormal IMD were associated with worse upper gut pain (*β* = 3.8, 95% CI: 2.1–5.4, *p* < 0.001), heartburn (*β* = 2.8, 95% CI: 1.4–4.2, *p* < 0.001), stomach burn (*β* = 1.8, 95% CI: 0.5–3.2, *p* = 0.009), and excessive fullness (*β* = 2.7, 95% CI: 1.0–4.4, *p* = 0.002).

**TABLE 2:** General linear model with interaction term between accommodation status and Gastric Alimetry phenotype for Total Symptom Burden.

	Beta	95% CI	*p*
Gastric Alimetry phenotype			
Normal	—	—	
Delayed meal response	2.4	−6.2, 11	0.6
High frequency	−0.53	−17, 16	> 0.9
Neuromuscular	−2.5	−11, 6.5	0.6
Intragastric meal distribution			
Normal	—	—	
Abnormal	−17	−23, −10	< 0.001
Phenotype × Accommodation			
Delayed meal response × Abnormal meal distribution	11	1.9, 20	0.017
High frequency × Abnormal meal distribution	19	2.1, 35	0.027
Neuromuscular × Abnormal meal distribution	12	2.3, 21	0.014

Abbreviation: CI, confidence interval.

## 4 Discussion

This study applied multimodal physiological testing to patients with chronic gastroduodenal symptoms. We found that IMD was associated with differences in gastric emptying and postprandial motor responses on BSGM, also correlating with a higher dyspeptic symptom burden. Proximal gastric retention was associated with delayed emptying, and in the setting of abnormal gastric neuromuscular function, delayed meal responses. Early maldistribution of food also most impacted those with abnormal spectrograms, indicating neuromuscular pathology, with antral predominant meal distribution inducing earlier postprandial antral motor responses that correlated with worse postprandial symptoms. Earlier movement of food to the antrum also had an apparent chronotropic effect, with gastric frequency showing a mild negative correlation to IMD0. Notably, stomach motility was robust to aberrant measures of meal distribution when neuromuscular function was intact (normal Gastric Alimetry spectrograms).

It has long been acknowledged that impaired fundic accommodation [8], and impaired postprandial antral motor activity are features of chronic gastroduodenal disorders [28, 29, 30]. Troncon et al. [8] first demonstrated the relationship between impaired fundic accommodation and earlier redistribution of food to the antrum in patients with functional dyspepsia, suggesting it is the resultant premature luminal distension of the antrum that causes symptoms of bloating and fullness. The multimodal noninvasive physiological testing applied here supports these findings in individuals with underlying gastric neuromuscular dysfunction, which confers reduced resilience to antral distension from impaired accommodation, causing dyspeptic symptoms and simultaneously altering the gastric meal response. However, it should be noted that the relationship between IMD measures and fundic accommodation requires further research, owing to the lack of reliable correlations to volumetric measures of accommodation on single photon emission computed tomography [31].

BSGM biomarkers have been refined to robustly characterize the myoelectrical activity that underpins the postprandial motor response of the stomach [32]. The PGF metric characterizes the frequency of the slow waves which coordinate gastric motility, while the GA‐RI characterizes the stability of this activity [22]. Additionally, the postprandial increase in BMI‐adjusted amplitude quantifies the strength of the underlying myoelectrical activity and coupled contractions [11, 22]. Together this suite of noninvasive biomarkers offers a comprehensive overview of the neuromuscular function of the stomach, offering additional insights to other physiological tests such as scintigraphy and IMD.

Several factors influence slow wave chronotropy [1], importantly, including stretch. Mechanosensitivity in interstitial cells of Cajal [33], results in increased slow wave frequencies with distension, as supported in the present study by the tendency toward higher gastric frequencies with more antral meal distributions (Figure 3). Excessive stretch, particularly in a neuromuscularly impaired stomach, may also predispose to decoupling of longitudinally propagating slow waves, impeding transit and inducing gastroduodenal symptoms [34]. This has been hypothesized to occur in the setting of reduced myenteric plexus CD206+ macrophages, which are known to drive ICC depletion in chronic gastroduodenal disorders [35]. BSGM therefore offers noninvasive biomarkers of gastric motor function, here emphasizing the clinical and physiological sequelae of fundic motor dysfunctions to the mechanosensitive ICC syncytium of the body and antrum [36, 37].

This multicentre study used simultaneous scintigraphic and noninvasive BSGM in patients with chronic gastroduodenal symptoms. Strengths of this study included consistent IMD evaluation by a single author across the multiinstitutional dataset and simultaneous multimodal testing. However, this study has some limitations. First, some subgroups had a low sample size, but where possible, correlations were assessed to minimize dichotomized analysis. In future, larger studies, particularly in patients with abnormal IMD, are desirable. Second, determination of abnormal IMD is based on a consensus‐based cut‐offs of the IMD analysis, which have not yet been externally validated [6]. Further physiological validation using barostat or single photon emission computed tomography may offer added insights on the impacts of gastric accommodation. Third, we use an egg meal that is standard for scintigraphy assessments, which is known to produce lower amplitudes and GA‐RI measurements compared to the higher calorie test meal standardly used for BSGM [37].

In extension to the present investigation focused on gastric motility evaluation, additionally, the influence of cellular neuromuscular pathology on these diverse gastric motor functions could be further elucidated. Directions for future work include better characterizing the role of duodenal feedback, particularly in the context of established association between dyspeptic symptoms and duodenal eosinophilia [38]. Similarly, visceral hypersensitivity, a disease mechanism particularly implicated in functional dyspepsia, could be better characterized in dedicated studies of afferent sensory function in response to gastric motor activity. Additionally, we acknowledge that gastric emptying and BSGM testing applied here do not evaluate inflammation, a disease mechanism that may be important in these cohorts. Finally, the therapeutic significance of paired myoelectrical and meal distribution abnormalities remains to be evaluated, with future therapeutic trials awaited.

In conclusion, multimodal physiological testing of gastric function shows that proximal gastric retention is associated with both delayed emptying and delayed postprandial motility responses on BSGM. Abnormal meal distribution had the most significant clinical impacts when myoelectrical abnormalities were present, with multiple motor dysfunctions on these tests being associated with worse dyspeptic symptoms. The addition of Gastric Alimetry BSGM biomarkers to standard functional assessment of the stomach aids in identifying gastric motility abnormalities.

## Author Contributions

C.V., A.A.G., H.P.P., and G.O. conceived the study. D.F., S.C., T.A., H.P.P. collected the data. C.V., A.A.G., G.S., S.C., A.H.M., R.A. analysed the data. V.H., C.N.A., A.H.M., T.A., H.P.P., G.O. provided supervision. G.O. and H.P.P. are the overall guarantor.

## Conflicts of Interest

A.A.G., C.N.A., T.A., and G.O. hold grants and intellectual property in the field of GI electrophysiology. G.O., A.A.G., G.S., S.C., C.N.A., D.F., and C.V. are members of the University of Auckland spin‐out companies: The Insides Company (G.O.), and Alimetry (A.G., D.F., G.S., S.C., C.V., C.N.A., and G.O.). A.H.M., T.A., H.P.P., and R.A. declare no conflicts of interest.

## Supporting information


**Data S1:** nmo70170‐sup‐0001‐DataS1.docx.

## Data Availability

The data that support the findings of this study are available on request from the corresponding author. The data are not publicly available due to privacy or ethical restrictions.
