# Supplementary material for: Relationship Between Intragastric Meal Distribution, Gastric Emptying, and Gastric Neuromuscular Dysfunction in Chronic Gastroduodenal Disorders
Source: Neurogastroenterol Motil. 2025 Sep 22;38(1):e70170. doi: 10.1111/nmo.70170 (PMC12814998; doi:10.1111/nmo.70170)
Supplement: Supplementary file 1 — Data S1: nmo70170‐sup‐0001‐DataS1.docx. [file NMO-38-e70170-s001.docx]

**Supplementary Appendix**

**Figure S1:** Intragastric meal distribution by Gastric Alimetry phenotype

**
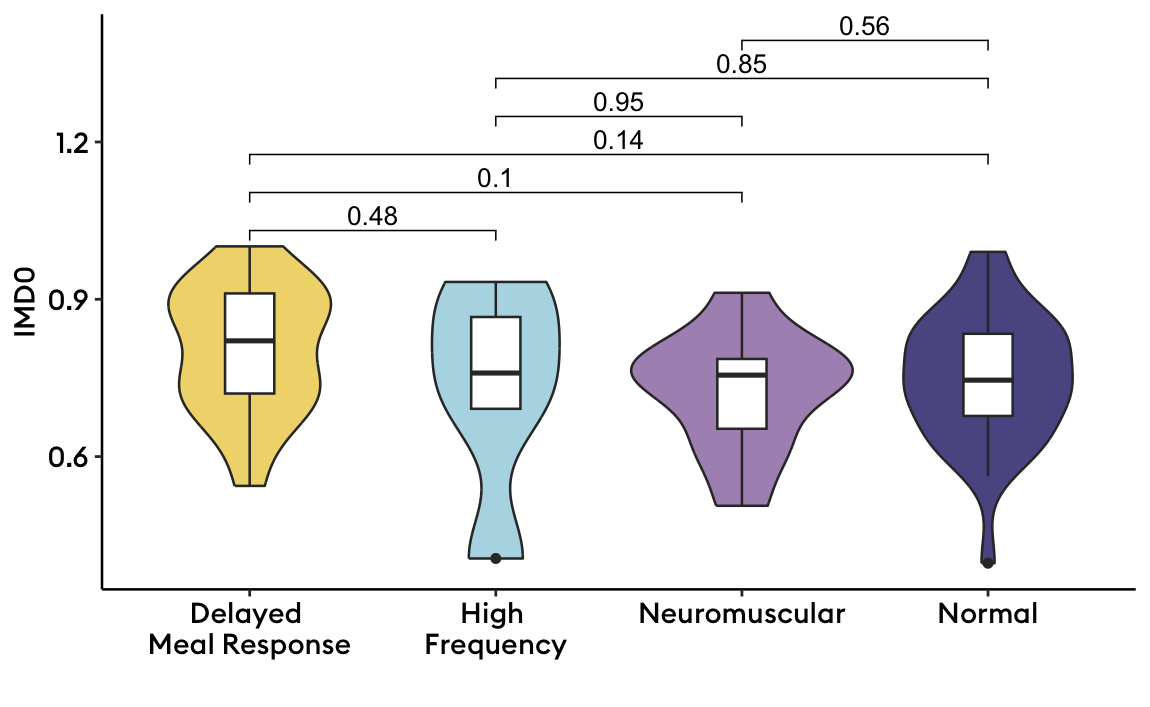
**

**Figure S2:** Correlation matrix between gastric emptying scintigraphy and body surface gastric metrics, and patient-reported symptoms and quality of life

**Table S1**: Physiological parameters stratified by gastric emptying status

|  |  | **Delayed gastric emptying** | **Normal gastric emptying** | **Total** | **p** |
| --- | --- | --- | --- | --- | --- |
| **IMD0 %** | Median (IQR) | 0.9 (0.8 to 0.9) | 0.7 (0.6 to 0.8) | 0.8 (0.7 to 0.9) | 0.004 |
| **T1/2** | Median (IQR) | 119.0 (87.4 to 157.3) | 46.0 (34.5 to 58.6) | 54.5 (37.6 to 89.4) | <0.001 |
| **Percentage retained at 4 h** | Median (IQR) | 18.9 (13.9 to 29.5) | 1.2 (0.4 to 3.1) | 3.0 (0.7 to 10.9) | <0.001 |
| **GA-RI** | Median (IQR) | 0.6 (0.5 to 0.6) | 0.5 (0.3 to 0.6) | 0.5 (0.3 to 0.6) | 0.044 |
| **PGF** | Median (IQR) | 3.0 (3.0 to 3.2) | 3.1 (2.9 to 3.2) | 3.0 (2.9 to 3.2) | 0.982 |
| **BMI-Adjusted Amplitude** | Median (IQR) | 36.0 (32.5 to 49.1) | 33.5 (26.4 to 39.6) | 34.3 (27.7 to 41.4) | 0.054 |
| **Meal response ratio** | Median (IQR) | 1.0 (0.9 to 1.4) | 1.1 (1.0 to 1.3) | 1.1 (1.0 to 1.4) | 0.351 |
| **Gastric Alimetry phenotype** | Delayed meal response | 8 (44.4) | 10 (20.4) | 18 (26.9) | 0.145 |
|  | High frequency | 2 (11.1) | 3 (6.1) | 5 (7.5) |  |
|  | Neuromuscular | 1 (5.6) | 9 (18.4) | 10 (14.9) |  |
|  | Normal | 7 (38.9) | 27 (55.1) | 34 (50.7) |  |

IMD0, intragastric meal distribution at time = 0; GES, gastric emptying scintigraphy; GA-RI, Gastric Alimetry Rhythm Index; PGF, Principal Gastric Frequency; IQR, interquartile range.

**Table S2**: Patient-reported outcomes stratified by results of IMD_0_, gastric emptying scintigraphy and body surface gastric mapping results. Data are reported as median (interquartile range).

| **Patient-Reported Outcome** | **Intragastric Meal Distribution** | | | **Gastric Emptying Scintigraphy** | | | **Body Surface Gastric Mapping** | | | | |
| --- | --- | --- | --- | --- | --- | --- | --- | --- | --- | --- | --- |
|  | **Abnormal IMD** | **Normal IMD** | **p** | **Delayed emptying** | **Normal emptying** | **p** | **Delayed meal response** | **High frequency** | **Neuromuscular** | **Normal** | **p** |
| **PAGI-SYM** | 2.9 (2.8 to 3.0) | 2.7 (2.1 to 3.3) | 0.91 | 2.7 (1.9 to 3.1) | 2.7 (2.1 to 3.3) | 0.838 | 2.8 (2.3 to 3.3) | 3.0 (2.7 to 3.3) | 2.9 (2.5 to 3.0) | 2.4 (1.8 to 3.2) | 0.507 |
| **PAGI-QOL** | 2.8 (2.8 to 3.3) | 2.9 (2.3 to 3.7) | 0.879 | 3.0 (2.5 to 3.8) | 2.9 (2.4 to 3.6) | 0.811 | 3.0 (2.4 to 3.6) | 2.9 (2.8 to 3.9) | 3.1 (2.5 to 3.2) | 2.9 (2.4 to 3.7) | 0.941 |
| **GCSI** | 2.9 (2.8 to 3.1) | 2.9 (2.1 to 3.5) | 0.672 | 2.9 (2.5 to 3.5) | 2.9 (2.1 to 3.4) | 0.559 | 3.1 (2.5 to 3.2) | 3.4 (2.9 to 3.6) | 2.9 (2.6 to 3.4) | 2.6 (2.0 to 3.5) | 0.714 |
| **Total Symptom Burden Score** | 14.8 (7.5 to 18.9) | 22.3 (11.3 to 31.5) | 0.215 | 24.4 (10.9 to 38.8) | 21.5 (11.2 to 29.2) | 0.471 | 21.8 (14.1 to 30.9) | 23.6 (12.9 to 24.3) | 19.2 (12.1 to 28.6) | 18.6 (6.8 to 35.2) | 0.872 |
| **Nausea** | 2.4 (1.3 to 2.6) | 3.2 (0.6 to 5.7) | 0.321 | 4.1 (1.2 to 5.7) | 2.6 (0.5 to 5.4) | 0.314 | 3.2 (2.1 to 5.2) | 1.3 (0.0 to 4.6) | 3.7 (1.6 to 4.8) | 2.2 (0.1 to 6.8) | 0.908 |
| **Bloating** | 0.6 (0.5 to 1.7) | 2.0 (0.5 to 4.3) | 0.45 | 1.4 (0.1 to 4.7) | 2.0 (0.5 to 4.2) | 0.649 | 2.8 (1.5 to 5.7) | 2.6 (2.0 to 5.0) | 1.2 (0.7 to 3.3) | 1.6 (0.0 to 3.5) | 0.314 |
| **Upper Gut Pain** | 1.6 (0.8 to 2.4) | 3.5 (1.2 to 5.6) | 0.184 | 4.0 (1.1 to 5.5) | 2.9 (1.2 to 5.5) | 0.898 | 3.0 (1.0 to 4.7) | 2.4 (2.0 to 3.8) | 2.9 (1.8 to 5.0) | 3.9 (1.0 to 5.8) | 0.846 |
| **Heartburn** | 0.3 (0.0 to 0.5) | 0.5 (0.0 to 3.0) | 0.66 | 0.8 (0.0 to 3.5) | 0.2 (0.0 to 2.6) | 0.363 | 1.1 (0.1 to 3.5) | 0.0 (0.0 to 0.0) | 0.0 (0.0 to 0.5) | 0.4 (0.0 to 3.0) | 0.138 |
| **Stomach Burn** | 1.0 (0.5 to 1.6) | 0.4 (0.0 to 3.0) | 0.554 | 0.4 (0.0 to 1.6) | 0.5 (0.0 to 3.0) | 0.731 | 0.6 (0.0 to 2.2) | 0.0 (0.0 to 3.4) | 0.6 (0.0 to 2.5) | 0.4 (0.0 to 3.8) | 0.917 |
| **Excessive Fullness** | 3.3 (0.4 to 4.4) | 4.1 (2.2 to 7.0) | 0.282 | 4.8 (3.0 to 7.7) | 3.6 (1.3 to 5.9) | 0.106 | 5.4 (3.2 to 7.4) | 5.4 (4.1 to 6.0) | 3.4 (1.2 to 6.3) | 3.5 (0.4 to 5.6) | 0.22 |
| **Early Satiety** | 6.0 (0.0 to 6.0) | 5.0 (0.0 to 8.0) | 0.592 | 7.5 (0.0 to 8.0) | 4.0 (0.0 to 7.0) | 0.266 | 5.5 (1.0 to 8.0) | 7.0 (0.0 to 7.0) | 5.5 (2.2 to 6.8) | 3.5 (0.0 to 8.0) | 0.708 |

FA, fundic accommodation.
